# Mitochondrial Dynamics and Mitophagy in the 6-Hydroxydopamine Preclinical Model of Parkinson's Disease

**DOI:** 10.1155/2012/131058

**Published:** 2012-08-16

**Authors:** Maria F. Galindo, Maria E. Solesio, Sandra Atienzar-Aroca, Maria J. Zamora, Joaquín Jordán Bueso

**Affiliations:** ^1^Unidad de Neuropsicofarmacología Traslacional, Complejo Hospitalario Universitario de Albacete, C/Hermanos Falcó 37, 02006 Albacete, Spain; ^2^Grupo de Neurofarmacología, Departamento Ciencias Médicas, Facultad de Medicina de Albacete, Universidad Castilla-La Mancha, IDINE, 02006 Albacete, Spain

## Abstract

We discuss the participation of mitochondrial dynamics and autophagy in the 6-hydroxidopamine-induced Parkinson's disease model. The regulation of dynamic mitochondrial processes such as fusion, fission, and mitophagy has been shown to be an important mechanism controlling cellular fate. An imbalance in mitochondrial dynamics may contribute to both familial and sporadic neurodegenerative diseases including Parkinson's disease. With special attention we address the role of second messengers as the role of reactive oxygen species and the mitochondria as the headquarters of cell death. The role of molecular signaling pathways, for instance, the participation of Dynamin-related protein 1(Drp1), will also be addressed. Furthermore evidence demonstrates the therapeutic potential of small-molecule inhibitors of mitochondrial division in Parkinson's disease. For instance, pharmacological inhibition of Drp1, through treatment with the mitochondrial division inhibitor-1, results in the abrogation of mitochondrial fission and in a decrease of the number of autophagic cells. Deciphering the signaling cascades that underlie mitophagy triggered by 6-OHDA, as well as the mechanisms that determine the selectivity of this response, will help to better understand this process and may have impact on human treatment strategies of Parkinson's disease.

## 1. Introduction

Parkinson's disease (PD) is progressive neurodegenerative condition that is characterized by the presence of motor and nonmotor symptoms, of which the etiology remains poorly understood. Nevertheless, a broad range of studies conducted over the past few decades have collectively identified a number of molecular/cellular events that might underlie PD pathogenesis. In particular, the participation of mitochondrial-mediated pathways has provided tremendous insights into the molecular pathways underlying dopaminergic neurodegeneration. Mitochondria can be considered as headquarters where the cell controls signaling pathways that under some circumstances can lead to cell death [1, 2]. Mitochondrial membrane permeabilization is a critical event during apoptosis and represents the point of no return of this lethal process [3]. For instance, the permeabilization of the mitochondrial outer membrane (MOMP), which allows the release of mitochondrial death factors, facilitates or triggers different signaling cascades that ultimately cause the execution of cell death. In many PD experimental models, including the addition of parkinsonian neurotoxins to cell cultures, the participation of MOMP has been described, resulting in the release of cytochrome c from mitochondria [4].

In the past, mitochondria have been suggested to be filamentous, rigid, and static organelles incrusted into the cytosol with the only function of being the main source or energy to the cell in the form of ATP. In fact, mitochondria are dynamic and mobile organelles that constantly undergo membrane remodeling through repeated cycles of fusion and fission. In addition, regulated turnover occurs via a specialized lysosome-mediated degradation pathway known as ‘‘mitophagy,” a term originally coined by Lemasters [5].

6-Hydroxydopamine (6-OHDA), also known as oxidopamine or 2,4,5-trihydroxyphenethylamine (C_8_H_11_NO_3_), is a toxic oxidative metabolite of dopamine and is detected in the brains and urine of Parkinson's disease (PD) patients. It has been applied broadly to generate experimental models of Parkinson's disease. There is accumulating evidence from *in vitro* and *in vivo* studies, implicating cell death in the etiology of the 6-OHDA model of PD [6–9].

## 2. 6-OHDA and Mitochondrial Dynamics

The regulation of mitochondrial dynamics processes such as fusion, fission, and mitophagy, signifies an important mechanism controlling cellular fate [10]. Mitochondrial fission and fusion are antagonistic activities. Their fundamental roles are to create a compartment that is a connected conductor, which is able to mix its contents. Also, they function to have access to mtDNA and its products in order to be distributed to distant cellular destinations through transport via actin or microtubule networks. The importance of mitochondrial dynamics to cellular function is perhaps best appreciated in neurons. These postmitotic cells, particularly those with vast axonal field, require high energy to support their operations, which include the active transportation of components (including mitochondria) toward metabolically demanding synaptic terminals that are distally located.

An imbalance in mitochondrial dynamics may contribute to both familial and sporadic neurodegenerative diseases including PD [11–14]. Evidence exists suggesting that an amplification of fission events can cause pathogenesis of human PD. Stress stimuli that are used to study PD, such as rotenone [15], annonacin [16], and 6-OHDA [17], are capable of inducing mitochondrial fission. Also, human fibroblasts from PD patients exhibit elevated levels of fragmented mitochondria [18].

Evidence has been presented showing that tipping the equilibrium toward continuous mitochondrial fission can evoke a neurodegenerative cascade [19]. Intriguingly, inherited loss-of-function mutations of MFN2 or OPA1 cause progressive neuropathies in humans. MFN2 mutations cause Charcot-Marie-Tooth type 2A (CMT-2A), a peripheral neuropathy characterized by motor and sensory neuron loss [20]. OPA1 mutations cause autosomal dominant optic atrophy, which is characterized by retinal ganglion cell and optic nerve degeneration [21].

In the dopaminergic cell line SH-SY5Y, using immunofluorescence studies with antibodies raised against the mitochondrial matrix protein MnSOD, we have shown that in untreated cells mitochondria exhibited a predominantly elongated and filamentous morphology. Strikingly, after addition of 50 *μ*M 6-OHDA mitochondria formed short and spherical structures, due to the fragmentation of single filamentous mitochondria into multiple isolated organelles [17]. Furthermore, time-lapse fluorescence microscopy revealed that 6-OHDA-induced mitochondrial fragmentation occurred rapidly and synchronous within 15 min after 6-OHDA addition and was visible in approximately 80% of the cells after 3 h. Thus, mitochondrial fragmentation appears to be an early event in 6-OHDA-induced cell death. Nevertheless, significant changes in the chromatin structure were not detected early on. 6-OHDA (50 *μ*M) had to be present more than 9 h to initiate significant changes in mitochondrial membrane potential in SH-SY5Y cells, placing mitochondrial alterations in an early stage of 6-OHDA-activated pathways.

Mitochondrial fission is highly regulated process and is mediated by a defined set of proteins [22–25]. One of these proteins, Dynamin-related protein 1 (Drp1), is a member of the dynamin family of large GTPases and mediates the scission of mitochondrial membranes through GTP hydrolysis. Drp1 predominantly is a cytoplasmic protein and associates with mitochondrial fission sites upon oligomerization [26, 27]. How Drp1 mediates outer membrane scission is unclear but it has been proposed that, similar to Dynamin, it may act as a mechanoenzyme [28]. Upon activation by an unknown mechanism, Drp1 assembles via Fis1 [29] into large complexes at future scission sites (cut sites) on the inner mitochondrial membrane [30]. Overexpression of Fis1 can induce directly both mitochondrial fragmentation and apoptosis [29]. However, Fis1 or mitochondrial fission is not requisites for apoptosis since cytochrome c release is prevented in cells overexpressing Fis1 when proapoptotic Bax/Bak are inactivated [31, 32].

Indicating that mitochondrial fission process may be important for apoptosis, dominant-negative forms of Drp1 that antagonize mitochondrial division delay the release of cytochrome c and the onset of cell death [33], although, not as potently as some antiapoptotic Bcl-2 family members, such as Bcl-xL. Moreover, ectopic Mfn2, Opa1, and mutant forms of Opa1 can also confer protection against programmed cell death [34–36].

We have revealed that 6-OHDA requires the dynamin-like GTPase Drp1 to induce mitochondrial division. We have also observed that Drp1 translocated to mitochondria 3 h after addition of 50 *μ*M 6-OHDA, although the levels of total Drp1 were unchanged in cellular extracts [37]. When SH-SY5Y cells were transfected with Drp1 siRNA duplexes to silence Drp1, 6-OHDA-induced mitochondrial fragmentation was inhibited. Furthermore, 6-OHDA-induced cell death was reduced after silencing of Drp1. In line with these findings, inhibition of Drp1 function in other experimental models has also been shown to prevent mitochondrial fission and cell death [33, 38, 39]. In a recent report, a block in mitochondrial fission by the expression of dominant-negative Drp1 or wild-type Mfn1 prevented mitochondrial fragmentation and rescued neurons from nitric- oxide- (NO-) induced degeneration and cell death.

Nowadays we have the pharmacological possibility of inhibiting Drp1 activity. For instance, Cassidy-Stone and colleagues [40] have identified an inhibitor of mitochondrial division, called mitochondrial division inhibitor-1 (mdivi-1), using yeast screens of chemical libraries. Mdivi-1 reduces mitochondrial fission after several insults [40, 41]. Mdivi-1 inhibits Dnm1 assembly and GTPase activity *in vitro*. Examining the activity of a series of mdivi-1 analogs shows a correlation between the degree of inhibition of GTPase activity and the extent of inhibition of yeast mitochondrial fission. Recently, another group identified an inhibitor of Dynamin-1, Dynamin-2, and DRP1, called Dynasore, which binds the GTPase domain and inhibits GTPase activity [42]. Mdivi-1 appears to be more selective than Dynasore, as it affects neither the activity of the Dynamin-1 GTPase *in vitro* nor that of the two mitochondrial dynamin family members mediating yeast mitochondrial fusion, Fzo1 or Mgm1. This is because mdivi-1 does not inhibit mitochondrial fusion *in vivo*. This specificity has been proposed to stem from mdivi-1 binding outside the GTPase domain to a surface that is involved in oligomeric assembly, thereby inhibiting Dnm1/DRP1 GTPase activation. Mechanistically, mdivi-1 acts as a mixed-type inhibitor to attenuate the early stages of division DRP assembly by preventing the polymerization of higher-order structures. Mdivi-1 selectively targets the unassembled pool of the mitochondrial division dynamin, and its binding creates and/or stabilizes an assembly-deficient conformation [43]. Furthermore, inhibiting mitochondrial division with mdivi-1 in Parkinson's disease cell culture models or a dominant negative form of Drp1 in Alzheimer's and Huntington's disease cell culture models attenuates disease-associated phenotypes [44]. For a review see [43].

Alternatively, mitochondrial dynamics may be initiated by insertion of the protooncogene Bcl-2 family into the MOM. The Bcl-2 family is composed of about 25 key regulators of apoptotic processes. These proteins are structural and functional homologs of the nematode protein CED-9 and are localized in the mitochondrial membrane. They contain up to four regions with a high homology to Bcl-2 (regions BH 1 to 4) [45]. Members containing only the BH3 region are proapoptotic proteins, and among them are Bax (X Bcl-2-associated protein), Bak (Bcl-2-antagonist/killer), BIM, and BID. Inactive Bax resides in the cytosol or is anchored to the laxly face of the membranes of various organelles [46]. Recently, several members of Bcl-2 family, including both, pro- and antiapoptotic proteins, have been shown to play a role in mitochondrial morphogenesis in healthy cells [37, 47]. Finding that Bax and Bak promote mitochondrial fusion in healthy cells [47] was unanticipated, as Bax and Bak form foci that colocalize with ectopic Mfn2 and Drp1 at the sites of mitochondrial division to promote mitochondrial fission during apoptosis [47]. After a cell death signal, the Bax protein acquires a homooligomeric shape and is incorporated into the outer mitochondrial membrane. Postmortem studies indicated that the presence of Bax and its translocation to the outer mitochondrial membrane may contribute to the death of dopaminergic neurons in PD [48]. In addition, the proapoptotic Bax protein colocalized to scission sites on mitochondria, suggesting that the mitochondrial fission machinery cooperates with the cell death machinery [49]. We have shown that Bax actively participates in the 6-OHDA preclinical model of PD [4, 17]. Furthermore, in our experimental model, mitochondrial Bax translocation took place after mitochondrial fragmentation and Drp1 translocation. SH-SY5Y cells consistently showed mitochondrial Bax localization 6 h after 6-OHDA addition. We were unable to find mitochondrial Bax-aggregation loci at the very early time points where mitochondrial fragmentation was already evident (<3 h of treatment). On the other hand, 6-OHDA-induced mitochondrial Bax translocation was independent of Drp1 and mitochondrial fission. Thus, Mdivi-1 failed to abrogate the translocation of Bax to the mitochondria upon 6-OHDA additions. In agreement with this, in Drp1^−/−^ cells [50] or in cells that were transfected with a dominant negative allele, DrpK38A, that is defective in GTP binding [33, 39, 49, 51], Bax translocates to the mitochondria with kinetics similar to those observed in wild-type cells.

In addition, several studies have reported that preventing mitochondrial fission during apoptosis leads to a partial inhibition of cytochrome c release [33, 35, 36, 38, 51]. Mitochondrial fission is not required for cell death. However, this does not exclude that fragmentation of the mitochondrial network might potentiate cell death.

Mitochondrial dynamics has also been proposed to play a role in the quality control of the organelle. During a division event, functionally asymmetric daughter mitochondria with different membrane potentials can be produced. The functional daughter, which retains a high membrane potential, can refuse with the mitochondrial network, whereas the dysfunctional daughter cannot refuse due to the low membrane potential and is subsequently flagged for autophagic degradation [52, 53].

## 3. 6-OHDA Inductors of Autophagy

The mitochondrial quality control hypothesis postulates that dysfunctional mitochondria are susceptible to degradation [53]. Autophagy is a stress-induced catabolic process involving the lysosome (or, in yeast, the analogous vacuole), which is conserved in all eukaryotes [54, 55]. According to the different pathways by which cargo is delivered to the lysosome or vacuole, autophagy is divided into three main types: chaperone-mediated autophagy, microautophagy, and macroautophagy [56]. Among the three main forms of autophagy, macroautophagy is the most widely studied and best characterized process. Macroautophagy, hereafter referred to as autophagy, is characterized by the formation of a cytosolic double-membrane vesicle, the autophagosome. During autophagy, cytoplasmic proteins, organelles or other materials are surrounded by phagophores, which expand and close to form autophagosomes. These autophagosomes fuse with lysosomes (or vacuoles) to form autolysosomes, in which the cytoplasmic cargos are degraded by resident hydrolases. The resulting degradation products are then transported back into the cytosol through the activity of membrane permeases for reuse [57]. Although autophagy is generally considered to be nonspecific, there are many examples of selective autophagy, including mitophagy (for mitochondria), ribophagy (for ribosomes), pexophagy (for peroxisomes), and reticulophagy (for the endoplasmic reticulum, ER) [58].

**Figure 1:**
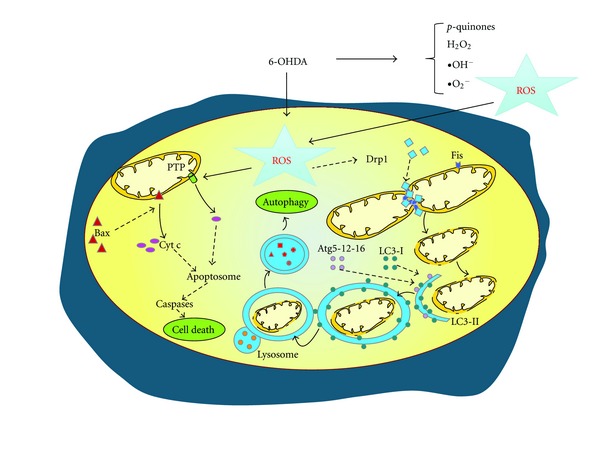
Mitochondrial fission and autophagy events are activated after 6-OHDA addition. Drp1 and Bax translocate from the cytosol to mitochondria. Drp1 has been proposed to encircle mitochondria to mediate constriction and this is followed by scission, which results in the formation of two separate mitochondria. Mitochondrial fission may play a role in the removal of dysfunctional mitochondria with reduced mitochondrial membrane potential, through an autophagy-lysosomal pathway named “mitophagy.”

The primary role of autophagy is to protect cells under stress conditions such as starvation. During periods of starvation, autophagy degrades cytoplasmic materials to produce amino acids and fatty acids that can be used to synthesize new proteins or are oxidized by mitochondria to produce ATP for cell survival [59]. However, when autophagy is excessively induced, it can result in autophagic cell death, so-called type II programmed cell death [60, 61] (Figure 1). In addition to stress management, autophagy is involved in normal development [60], senescence [62], lifespan extension [63], immunity, and defense against microbial invasion [64]. In particular, autophagy has been observed to be deregulated in PD brains [65]. Consistent with these observations, suppression of basal autophagy causes neurodegeneration in mice [66]. Moreover, rapamycin, a well-known autophagic inducer, protects from PD toxins [67].

Unfortunately, to date it remains unknown what the underlying mechanisms of autophagy are in terms of procell survival versus procell death effects. Therefore, mechanisms that underlie these dual functions of autophagy (cell survival and cell death) need to be explored in the future. There are several hypotheses. The procell death effect of autophagy could be related to the activation of apoptosis, which would imply that autophagy is an upstream event of apoptosis [68]. For the cytoprotective effect of autophagy against stress, one possible mechanism for autophagic cell death could involve the autophagic degradation of a negative effector of apoptosis. This is supported by a recent demonstration that autophagic degradation of the *Drosophila *inhibitor of apoptosis (IAP) dBruce controls apoptotic cell death in nurse cells during late *Drosophila melanogaster *oogenesis [68]. Alternatively, autophagic degradation of active caspase-8, a positive effector of apoptosis, may also be responsible for the inhibition of apoptotic cell death in mammalian cells [69].

Autophagy is induced by 6-OHDA treatment. The 6-OHDA-induced autophagy correlated with an increase in the LC3-II protein level and with the accumulation of autophagic vacuoles in the cytoplasm and the activation of lysosomes [70]. It remains to be determined whether the induction of autophagy by 6-OHDA is related to cell death or to a cytoprotective response, which is activated by dying cells in order to cope with stress. In a previous study, tyrosine hydrolase-positive neurons in substantia nigra were protected from 6-OHDA-induced cell death when they were pretreated with the autophagy inhibitor 3-methyladenine [70]. On the other hand, experiments using neuron-specific knockout mouse models have demonstrated that autophagy deficiency leads to protein aggregation and neurodegeneration, even in the absence of disease-related aggregate-prone proteins [58].

## 4. ROS as Second Messengers in 6-OHDA-Induced Pathways

Reactive oxygen species (ROS) are important for execution of physiological functions. However, excessive production of ROS is detrimental to the cell. Following an increase in ROS production, the cell's redox equilibrium is shifted to a more oxidized state, affecting both the structure and the function of different molecules. This may lead to specific toxic processes, which compromise the redox status of the cell and can cause cell death. Due to high levels of polyunsaturated fatty acids in their membranes and the relatively low activity of endogenous antioxidant enzymes, cells in the brain are particularly susceptible to oxidative damage.

On the other hand, ROS are able to induce pore opening [71]. Exposure of mitochondria to these species causes a decrease in the content of thiol residues in the membrane. It also leads to a collapse of the mitochondrial electrical transmembrane potential [72], which is prevented by the presence of antioxidant drugs like vitamin E and glutathione.

Under physiological conditions, 6-OHDA is rapidly and nonenzymatically oxidized by molecular oxygen to form 1,4-para-quinone and its degradation products [73], along with production of ROS such as hydrogen peroxide (H_2_O_2_), superoxide radical (O_2_^−^) and hydroxyl radical (^∙^OH^−^). Quinones react with nucleotic groups of macromolecules, leading to inactive or destroyed quinoproteins, which do not seem to contribute significantly to the observed cytotoxic effects of 6-OHDA. H_2_O_2_ can enter the cells and reacts with trace metals to form highly reactive ^∙^OH^−^ [74]. This can oxidatively damage proteins, lipids, and DNA [75]. We have shown that 6-OHDA concentrations that were nontoxic to cell cultures did not significantly increase H_2_O_2_ production [76]. Moreover, H_2_O_2_ addition to cultures produced a pattern of cell death similar to 6-OHDA.

In addition to the non-enzymatic self-auto-oxidation process, microinjection of 6-OHDA into the striatum may lead to the generation of H_2_O_2_ via a mitochondrial enzymatic oxidation process. Inhibition of complex I of the electron transport chain also stimulated mitochondrial production of superoxide radicals. These superoxide radicals were then catalyzed to H_2_O_2_ by superoxide dismutase and, subsequently, ^∙^OH^−^ may arise from the breakdown of H_2_O_2_. This may be associated with the mitochondrial dysfunction seen in our experiments, because ^∙^OH^−^ rapidly attacks other biological molecules. The radicals produced in molecules such as lipids and proteins may also interact with mitochondrial enzymes to cause degradation.

In the signaling pathways that are involved in 6-OHDA-induced mitochondrial fission and autophagy, evidence revealed a key role for ROS. Our data demonstrated a relationship between ROS and 6-OHDA-induced mitochondrial fission and, subsequently, mitophagy. Intriguingly, 6-OHDA increases H_2_O_2_ between the cells. We made this observation using the dye CM-H_2_DCFDA to measure peroxide-like formation. This specific tool allows us to ascertain the role of ROS in the mitochondrial dynamics process. Given that the inhibition of this dynamic process, using mdivi-1, did no block mitochondrial H_2_O_2_ production upon 6-OHDA treatments, H_2_O_2_ production is upstream of mitochondrial fission. In addition, TEMPOL and MnTBAP, two well-known antioxidant drugs, abolished translocation of Drp1 to mitochondria and, consequently, 6-OHDA-induced mitochondrial fission. In keeping with this interpretation, oxidative stress might be responsible for induced mitochondrial fission in several processes, including PD, perhaps due to a posttranslational redox change in the Drp1 protein [77, 78].

In addition, nitric oxide induces profound mitochondrial fission [44]. Cultured cerebrocortical neurons exposed to the physiological NO donor, S-nitrosocysteine, induced SNO-Drp1 formation and led to the accumulation of excessively fragmented mitochondria. SNO-Drp1-induced mitochondrial fragmentation caused synaptic damage, an early characteristic feature of AD and, subsequently, apoptotic neuronal cell death. Importantly, blockade of Drp1 nitrosylation (using the Drp1 (C644A) mutant) prevented A-*β*-mediated mitochondrial fission, synaptic loss, and neuronal cell death, suggesting that the posttranslational modification (S-nitrosylation) of Drp1 contributes to the pathogenesis of AD. Thus, SNO-Drp1 may represent a potential new therapeutic target for protecting neurons and their synapses in sporadic AD. Multiple groups have now reported on S-nitrosylation and subsequent activation of dynamin family members, including Drp1 [79–82].

In conclusion, although we await further clarifications on the role of mitochondrial fission and mitophagy in PD, we consider this pathway as a promising new and attractive pharmacological target. Interestingly, recent evidence has identified new molecules involved in PD such as Parkin and PINK1, key regulators of mitophagy.

## References

[1] Jordán J, Ceña V, Prehn JHM (2003). Mitochondrial control of neuron death and its role in neurodegenerative disorders. *Journal of Physiology and Biochemistry*.

[2] Jordan J, de Groot PWJ, Galindo MF (2011). Mitochondria: the headquarters in ischemia-induced neuronal death. *Central Nervous System Agents in Medicinal Chemistry*.

[3] Galluzzi L, Blomgren K, Kroemer G (2009). Mitochondrial membrane permeabilization in neuronal injury. *Nature Reviews Neuroscience*.

[4] Gomez-Lazaro M, Galindo MF, Concannon CG (2008). 6-Hydroxydopamine activates the mitochondrial apoptosis pathway through p38 MAPK-mediated, p53-independent activation of Bax and PUMA. *Journal of Neurochemistry*.

[5] Lemasters JJ (2005). Selective mitochondrial autophagy, or mitophagy, as a targeted defense against oxidative stress, mitochondrial dysfunction, and aging. *Rejuvenation Research*.

[6] Blum D, Torch S, Lambeng N (2001). Molecular pathways involved in the neurotoxicity of 6-OHDA, dopamine and MPTP: contribution to the apoptotic theory in Parkinson’s disease. *Progress in Neurobiology*.

[7] Bové J, Prou D, Perier C, Przedborski S (2005). Toxin-induced models of Parkinson’s disease. *NeuroRx*.

[8] Duty S, Jenner P (2011). Animal models of Parkinson's disease: a source of novel treatments and clues to the cause of the disease. *British Journal of Pharmacology*.

[9] Fernandez-Gomez FJ, Gomez-Lazaro M, Pastor D (2005). Minocycline fails to protect cerebellar granular cell cultures against malonate-induced cell death. *Neurobiology of Disease*.

[10] Liesa M, Palacín M, Zorzano A (2009). Mitochondrial dynamics in mammalian health and disease. *Physiological Reviews*.

[11] Cho DH, Nakamura T, Lipton SA (2010). Mitochondrial dynamics in cell death and neurodegeneration. *Cellular and Molecular Life Sciences*.

[12] Lees AJ, Hardy J, Revesz T (2009). Parkinson’s disease. *The Lancet*.

[13] Su B, Wang X, Zheng L, Perry G, Smith MA, Zhu X (2010). Abnormal mitochondrial dynamics and neurodegenerative diseases. *Biochimica et Biophysica Acta*.

[14] Thomas B, Beal MF (2007). Parkinson's disease. *Human Molecular Genetics*.

[15] De Palma C, Falcone S, Pisoni S (2010). Nitric oxide inhibition of Drp1-mediated mitochondrial fission is critical for myogenic differentiation. *Cell Death and Differentiation*.

[16] Escobar-Khondiker M, Höllerhage M, Muriel MP (2007). Annonacin, a natural mitochondrial complex I inhibitor, causes tau pathology in cultured neurons. *Journal of Neuroscience*.

[17] Gomez-Lazaro M, Bonekamp NA, Galindo MF, Jordán J, Schrader M (2008). 6-Hydroxydopamine (6-OHDA) induces Drp1-dependent mitochondrial fragmentation in SH-SY5Y cells. *Free Radical Biology and Medicine*.

[18] Exner N, Treske B, Paquet D (2007). Loss-of-function of human PINK1 results in mitochondrial pathology and can be rescued by parkin. *Journal of Neuroscience*.

[19] Bossy-Wetzel E, Barsoum MJ, Godzik A, Schwarzenbacher R, Lipton SA (2003). Mitochondrial fission in apoptosis, neurodegeneration and aging. *Current Opinion in Cell Biology*.

[20] Züchner S, Mersiyanova IV, Muglia M (2004). Mutations in the mitochondrial GTPase mitofusin 2 cause Charcot-Marie-Tooth neuropathy type 2A. *Nature Genetics*.

[21] Delettre C, Lenaers G, Griffoin JM (2000). Nuclear gene OPA1, encoding a mitochondrial dynamin-related protein, is mutated in dominant optic atrophy. *Nature Genetics*.

[22] Cerveny KL, McCaffery JM, Jensen RE (2001). Division of mitochondria requires a novel DNM1-interacting protein, Net2p. *Molecular Biology of the Cell*.

[23] Karbowski M, Jeong SY, Youle RJ (2004). Endophilin B1 is required for the maintenance of mitochondrial morphology. *Journal of Cell Biology*.

[24] Mozdy AD, McCaffery JM, Shaw JM (2000). Dnm1p GTPase-mediated mitochondrial fission is a multi-step process requiring the novel integral membrane component Fis1p. *Journal of Cell Biology*.

[25] Otsuga D, Keegan BR, Brisch E (1998). The dynamin-related GTPase, Dnm1p, controls mitochondrial morphology in yeast. *Journal of Cell Biology*.

[26] Labrousse AM, Zappaterra MD, Rube DA, Van der Bliek AM (1999). C. elegans dynamin-related protein DRP-1 controls severing of the mitochondrial outer membrane. *Molecular Cell*.

[27] Legesse-Miller A, Massol RH, Kirchhausen T (2003). Constriction and Dnm1p recruitment are distinct processes in mitochondrial fission. *Molecular Biology of the Cell*.

[28] Yoon Y, Pitts KR, McNiven MA (2001). Mammalian dynamin-like protein DLP1 tubulates membranes. *Molecular Biology of the Cell*.

[29] James DI, Parone PA, Mattenberger Y, Martinou JC (2003). hFis1, a novel component of the mammalian mitochondrial fission machinery. *Journal of Biological Chemistry*.

[30] Frank S, Robert EG, Youle RJ (2003). Scission, spores, and apoptosis: a proposal for the evolutionary origin of mitochondria in cell death induction. *Biochemical and Biophysical Research Communications*.

[31] Alirol E, James D, Huber D (2006). The mitochondrial fission protein hFis1 requires the endoplasmic reticulum gateway to induce apoptosis. *Molecular Biology of the Cell*.

[32] James TN, Terasaki F, Pavlovich ER, Vikhert AM (1993). Apoptosis and pleomorphic micromitochondriosis in the sinus nodes surgically excised from five patients with the long QT syndrome. *The Journal of Laboratory and Clinical Medicine*.

[33] Frank S, Gaume B, Bergmann-Leitner ES (2001). The role of dynamin-related protein 1, a mediator of mitochondrial fission, in apoptosis. *Developmental Cell*.

[34] Frezza C, Cipolat S, Martins de Brito O (2006). OPA1 controls apoptotic cristae remodeling independently from mitochondrial fusion. *Cell*.

[35] Neuspiel M, Zunino R, Gangaraju S, Rippstein P, McBride H (2005). Activated mitofusin 2 signals mitochondrial fusion, interferes with Bax activation, and reduces susceptibility to radical induced depolarization. *Journal of Biological Chemistry*.

[36] Sugioka R, Shimizu S, Tsujimoto Y (2004). Fzo1, a protein involved in mitochondrial fusion, inhibits apoptosis. *Journal of Biological Chemistry*.

[37] Solesio MS, Saez-Atienzar S, Jordan J, Galindo MF Characterization of mitophagy in the 6-hydoxydopamine Parkinson's disease model.

[38] Breckenridge DG, Stojanovic M, Marcellus RC, Shore GC (2003). Caspase cleavage product of BAP31 induces mitochondrial fission through endoplasmic reticulum calcium signals, enhancing cytochrome c release to the cytosol. *Journal of Cell Biology*.

[39] Germain M, Mathai JP, McBride HM, Shore GC (2005). Endoplasmic reticulum BIK initiates DRP1-regulated remodelling of mitochondrial cristae during apoptosis. *EMBO Journal*.

[40] Cassidy-Stone A, Chipuk JE, Ingerman E (2008). Chemical inhibition of the mitochondrial division dynamin reveals its role in Bax/Bak-dependent mitochondrial outer membrane permeabilization. *Developmental Cell*.

[41] Cereghetti GM, Stangherlin A, Martins De Brito O (2008). Dephosphorylation by calcineurin regulates translocation of Drp1 to mitochondria. *Proceedings of the National Academy of Sciences of the United States of America*.

[42] Macia E, Ehrlich M, Massol R, Boucrot E, Brunner C, Kirchhausen T (2006). Dynasore, a cell-permeable inhibitor of dynamin. *Developmental Cell*.

[43] Lackner LL, Nunnari J (2010). Small molecule inhibitors of mitochondrial division: tools that translate basic biological research into medicine. *Chemistry and Biology*.

[44] Barsoum MJ, Yuan H, Gerencser AA (2006). Nitric oxide-induced mitochondrial fission is regulated by dynamin-related GTPases in neurons. *EMBO Journal*.

[45] Reed JC (2006). Proapoptotic multidomain Bcl-2/Bax-family proteins: mechanisms, physiological roles, and therapeutic opportunities. *Cell Death and Differentiation*.

[46] Wolter KG, Hsu YT, Smith CL, Nechushtan A, Xi XG, Youle RJ (1997). Movement of Bax from the cytosol to mitochondria during apoptosis. *Journal of Cell Biology*.

[47] Karbowski M, Norris KL, Cleland MM, Jeong SY, Youle RJ (2006). Role of Bax and Bak in mitochondrial morphogenesis. *Nature*.

[48] Hartmann A, Michel PP, Troadec JD (2001). Is bax a mitochondrial mediator in apoptotic death of dopaminergic neurons in Parkinson’s disease?. *Journal of Neurochemistry*.

[49] Karbowski M, Lee YJ, Gaume B (2002). Spatial and temporal association of Bax with mitochondrial fission sites, Drp1, and Mfn2 during apoptosis. *Journal of Cell Biology*.

[50] Ishihara N, Nomura M, Jofuku A (2009). Mitochondrial fission factor Drp1 is essential for embryonic development and synapse formation in mice. *Nature Cell Biology*.

[51] Lee YJ, Jeong SY, Karbowski M, Smith CL, Youle RJ (2004). Roles of the mammalian mitochondrial fission and fusion mediators Fis1, Drp1, Opa1 in apoptosis. *Molecular Biology of the Cell*.

[52] Parone PA, Da Druz S, Tondera D (2008). Preventing mitochondrial fission impairs mitochondrial function and leads to loss of mitochondrial DNA. *PLoS ONE*.

[53] Twig G, Elorza A, Molina AJA (2008). Fission and selective fusion govern mitochondrial segregation and elimination by autophagy. *EMBO Journal*.

[54] Chen Y, Klionsky DJ (2011). The regulation of autophagy—unanswered questions. *Journal of Cell Science*.

[55] Esclatine A, Chaumorcel M, Codogno P (2009). Macroautophagy signaling and regulation. *Current Topics in Microbiology and Immunology*.

[56] Klionsky DJ (2005). The molecular machinery of autophagy: unanswered questions. *Journal of Cell Science*.

[57] Klionsky DJ (2007). Autophagy: from phenomenology to molecular understanding in less than a decade. *Nature Reviews Molecular Cell Biology*.

[58] He C, Klionsky DJ (2006). Autophagy and neurodegeneration.. *ACS chemical biology*.

[59] Levine B, Yuan J (2005). Autophagy in cell death: an innocent convict?. *Journal of Clinical Investigation*.

[60] Levine B, Klionsky DJ (2004). Development by self-digestion: molecular mechanisms and biological functions of autophagy. *Developmental Cell*.

[61] Maiuri MC, Zalckvar E, Kimchi A, Kroemer G (2007). Self-eating and self-killing: crosstalk between autophagy and apoptosis. *Nature Reviews Molecular Cell Biology*.

[62] Young ARJ, Narita M, Ferreira M (2009). Autophagy mediates the mitotic senescence transition. *Genes and Development*.

[63] Vellai T, Takács-Vellai K, Sass M, Klionsky DJ (2009). The regulation of aging: does autophagy underlie longevity?. *Trends in Cell Biology*.

[64] Deretic V, Levine B (2009). Autophagy, immunity, and microbial adaptations. *Cell Host and Microbe*.

[65] Chu Y, Dodiya H, Aebischer P, Olanow CW, Kordower JH (2009). Alterations in lysosomal and proteasomal markers in Parkinson’s disease: relationship to alpha-synuclein inclusions. *Neurobiology of Disease*.

[66] Hara T, Nakamura K, Matsui M (2006). Suppression of basal autophagy in neural cells causes neurodegenerative disease in mice. *Nature*.

[67] Malagelada C, Jin ZH, Jackson-Lewis V, Przedborski S, Greene LA (2010). Rapamycin protects against neuron death in in vitro and in vivo models of Parkinson’s disease. *Journal of Neuroscience*.

[68] Nezis IP, Shravage BV, Sagona AP (2010). Autophagic degradation of dBruce controls DNA fragmentation in nurse cells during late Drosophila melanogaster oogenesis. *Journal of Cell Biology*.

[69] Hou W, Han J, Lu C, Goldstein LA, Rabinowich H (2010). Autophagic degradation of active caspase-8: a crosstalk mechanism between autophagy and apoptosis. *Autophagy*.

[70] Li L, Wang X, Fei X, Xia L, Qin Z, Liang Z (2011). Parkinson’s disease involves autophagy and abnormal distribution of cathepsin L. *Neuroscience Letters*.

[71] Jordán J, Galindo MF, Tornero D (2002). Superoxide anions mediate veratridine-induced cytochrome c release and caspase activity in bovine chromaffin cells. *British Journal of Pharmacology*.

[72] Sakurai K, Stoyanovsky DA, Fujimoto Y, Cederbaum AI (2000). Mitochondrial permeability transition induced by 1-hydroxyethyl radical. *Free Radical Biology and Medicine*.

[73] Gee P, Davison AJ (1989). Intermediates in the aerobic autoxidation of 6-hydroxydopamine: relative importance under different reaction conditions. *Free Radical Biology and Medicine*.

[74] Koppenol WH (2001). The Haber-Weiss cycle—70 years later. *Redox Report*.

[75] Beckman KB, Ames BN (1997). Oxidative decay of DNA. *Journal of Biological Chemistry*.

[76] Galindo MF, Jordán J, González-García C, Ceña V (2003). Chromaffin cell death induced by 6-hydroxydopamine is independent of mitochondrial swelling and caspase activation. *Journal of Neurochemistry*.

[77] Nakamura T, Lipton SA (2010). Redox regulation of mitochondrial fission, protein misfolding, synaptic damage, and neuronal cell death: potential implications for Alzheimer’s and Parkinson’s diseases. *Apoptosis*.

[78] Nakamura T, Lipton SA (2011). Redox modulation by S-nitrosylation contributes to protein misfolding, mitochondrial dynamics, and neuronal synaptic damage in neurodegenerative diseases. *Cell Death and Differentiation*.

[79] Cho DH, Nakamura T, Fang J (2009). *β*-Amyloid-related mitochondrial fission and neuronal injury. *Science*.

[80] Kang-Decker N, Cao S, Chatterjee S (2007). Nitric oxide promotes endothelial cell survival signaling through S-nitrosylation and activation of dynamin-2. *Journal of Cell Science*.

[81] Wang G, Moniri NH, Ozawa K, Stamler JS, Daaka Y (2006). Nitric oxide regulates endocytosis by S-nitrosylation of dynamin. *Proceedings of the National Academy of Sciences of the United States of America*.

[82] Wang X, Su B, Lee HG (2009). Impaired balance of mitochondrial fission and fusion in Alzheimer’s disease. *Journal of Neuroscience*.

